# Caries risk factors among Indonesian adults: insights from the national health survey

**DOI:** 10.1590/1807-3107bor-2025.vol39.025

**Published:** 2025-02-24

**Authors:** Yuanita Lely RACHMAWATI, Agustine Hanafi PUTRI, Rahmavidyanti PRIYANTO, Khansa Catraliya NABILAH, Ananda Dhea Salsabila GANI, Tuti Ningseh MOHD-DOM

**Affiliations:** (a) Universitas Brawijaya, Faculty of Dentistry, Department of Community and Preventive Dentistry, Malang, Indonesia.; (b) Universitas Brawijaya, Faculty of Dentistry, Department of Oral Medicine, Malang, Indonesia.; (c) Universitas Brawijaya, Faculty of Dentistry, Department of Conservative and Endodontics, Malang, Indonesia.; (d) Universitas Brawijaya, Faculty of Dentistry, Malang, Indonesia.; (e) Universiti Kebangsaan Malaysia, Faculty of Dentistry, Department of Family Oral Health, Kuala Lumpur, Malaysia.

**Keywords:** Dental Caries, Adult, Risk Factors, Epidemiology

## Abstract

Although caries is a preventable disease, it is still a health burden in all countries in all age categories. This study aimed to identify the determinants associated with caries risk factors in Indonesian adults. This secondary analysis used 2018 National Health Survey data, which included 37,057 respondents aged ≥ 15 years. The sampling design was based on census blocks selected using linear systematic sampling with probability proportional to size (PPS). The survey included an interview to collect data on sociodemographic characteristics, oral health practice, and health behavior. An oral examination was conducted to measure the DMFT (decayed, missing, filled- teeth) index. Multivariable models were generated to estimate the odds ratio (OR) and confidence intervals (95% CI) using caries as the dependent variable. The highest average DMFT index was seen in those respondents aged over 45 years, those from rural areas, individuals with low formal education, those who consumed sweets and sugary beverages at least once a day, those who did not brush their teeth regularly, and those who smoked. The multivariable analysis showed the potential risk of caries: risk of 19.51 times for older adults, 74% for male, 94% for those living in rural areas, 1.62 times for those with lower formal education, 78% for those with no dental visits, 1.18 times for those who eat sweets often, 1.37 times for those who drink sugary beverages often, 1.48 times for those who do not brush their teeth regularly, and 1.3 times for those who smoke. The multifaceted nature of caries in the Indonesian adult population highlights the influence of various factors, ranging from lifestyle habits and oral hygiene practices to access to dental services, age, and educational level.

## Introduction

While dental caries is a preventable disease, it continues to pose a significant global health burden across all age groups. Many countries have conducted national surveys on caries prevalence and revealed its widespread impact. The national survey in Chile showed that caries in individuals aged 35 years and over had an average DMFT of 15.06,^1^ while both Norway and Turkey reported an average DMFT of 10.8.^2,3^ A national survey in Ethiopia showed a caries prevalence of 60% in the adult population, among which 53% had one or more untreated carious lesions.^3^ Reports from Spain stated a 92% prevalence of caries in the adult population with a DMFT index of 7.64.^4^ These surveys reported substantial prevalence rates and high DMFT indices, indicating the severity of this condition.

In Indonesia, a country with a population exceeding 280 million, the burden of dental caries is particularly significant. The 2018 national health survey reported a caries prevalence of 88.8%, with the highest prevalence (over 92.2%) observed in individuals aged 5-9 years and 35 years and over. Moreover, the national DMFT index was reported to be 7.1. A high DMFT index (6.9) was found in the 35–44 year age group and 16.8 for individuals aged 65 years and over. Comparatively, the earlier 2013 national survey reported an average DMFT of 4.6, with an index of 5.4 for ages 35–44, and 18.9 for ages ≥ 65 years, indicating that the caries index has not decreased over the years.^5^

The implications of untreated caries are far-reaching, affecting various aspects of human life. The impact is significant, ranging from worse quality of life caused by impaired chewing function, speech difficulties, and reduced self-confidence, to economic implications such as lost workdays due to toothache. ^6^ Both the growth and development of children are also affected by inadequate nutritional intake resulting from impaired chewing function.^9^ Studies have shown an association between dental caries and systemic conditions such as diabetes mellitus and cardiovascular diseases.^10^ Additionally, there is increased risk of delivering low-birthweight or large-for-gestational-age infants. ^11^

Dental caries is a complex disease in which there exists an interplay of three important elements: the tooth surface, cariogenic bacteria, and fermentable carbohydrates. However, the development of caries also depends on social, behavioral, and environmental factors.^12^ Several studies have identified risk factors for caries. In Ethiopia, research identified various factors contributing to caries prevalence, including area of residence, educational level, dental attendance, frequency of tooth cleaning, sugar consumption, tooth cleaning material, toothpaste use, and khat chewing frequency.^3^ Meanwhile, in New South Wales, brushing one’s teeth twice or more per day, access to fluoridated water supply, consumption of one or more sugary drinks per day, and family income have been identified as significant determinants of dental health.^13^

While these findings have identified risk factors for dental caries, the determinants may differ from one country to another due to cultural differences, lifestyle, economic conditions, and sociodemographic characteristics. Therefore, it is crucial to conduct a study to identify the determinants that act as risk factors for dental caries in Indonesia to implement a national prevention strategy.

Several studies on caries and its risk factors in Indonesian adults have been published but with a limited population in one province and only a few adult age categories. In Indonesian adults aged 20–39 years in Semarang City , dental caries was found to be influenced by risk factors such as tooth structure, saliva pH, plaque index and sugar consumption.^14^ While in Jakarta city, the prevalence of caries in respondents aged 34–44 years was influenced by educational level, occupation, sugar consumption, and smoking.^15^

This study aims to analyze the determinants of caries occurrence by examining sociodemographic factors, oral health practices, and general health behaviors in the adult population. The data are nationally representative, sourced from the 2018 Indonesian National Health Survey. This research hypothesizes that sociodemographic factors, oral health practice, and general health behavior would influence the occurrence of caries in Indonesian adults. By identifying these determinants, we hope to provide the government with valuable insights to help them design national oral health programs to reduce the prevalence of dental caries in Indonesia.

## Methods

### Study design

This study employs secondary data from the 2018 National Health Survey, accessible through the Indonesian Health Development Policy Agency, following specific requirements and procedures outlined at www.badankebijakan.kemkes.go.id. The 2018 National Health Survey is a cross-sectional study approved by the Ethics Committee of Health Research, Faculty of Medicine, Universitas Islam Negeri Maulana Malik Ibrahim Malang (process no. 21/EC/KEPK-FKIK/40/VIII/2023). This research report followed the Strengthening the Reporting of Observational Studies in Epidemiology (STROBE) guidelines for cross-sectional studies.^16^

### Participants

The survey targeted 30,000 census blocks from the National Socioeconomic Survey conducted by the Central Bureau of Statistics. A two-stage sampling method was employed, using the probability proportional to size method and systematic linear sampling. The first stage involved implicit stratification of all census blocks from the 2010 Population Census, based on welfare strata. The second stage involved selecting 10 households in each census block, updated by systematic sampling with implicit stratification based on the highest educational level completed by the household head, to maintain the diversity of household characteristics. The study included residents aged 15 years and older, but excluded those with a complete loss of all-natural teeth. The analysis included data from 37,057 respondents.^17^ Age categorization followed the age standard of the Australian Bureau of Statistics, namely 15–24; 25–44; 45–64; and > 64.^18^

### Data collection and variables

The 2018 National Health Survey data collection was carried out through interviews and oral examinations. The interviews were conducted using a guided questionnaire to explore sociodemographic and behavioral data. The sociodemographic data collected included the area of residence (urban or rural), sex (male or female), age, and educational level, while behavioral data included oral health practice (consumption of sugary beverages and sweets, daily toothbrushing, visits to a dental professional in the past year), and general health behavior (smoking).^19^

The oral examination was performed using the WHO-approved form and protocol for the DMFT index. Instruments for oral examination were plane mouth mirrors, metallic periodontal probes (community periodontal index (CPI) probes that conform to WHO specifications), and several pairs of tweezers. In addition, the examiner also used a small cotton swab to dry the tooth surface and remove food debris, if necessary. The DMFT index was used to quantify the number of coronal caries experienced based on the following categories: DT (decayed tooth), MT (missing tooth), and FT (filled tooth) due to caries.^19^ Before data collection, all participants signed an informed consent form.

Recruitment, selection, and training were conducted by the National Health Survey committee for interviewers and examiners to ensure data quality. The interviewer had at least a diploma in health science, and the examiner of oral conditions was a dentist. The Indonesian Health Researchers Association conducted independent external validation and calibrated the examiners, also measuring inter-examiner reliability, with a Kappa value greater than 0.9 for the oral examination.^17^

### Statistical analysis

Data analysis was conducted using SPSS Statistical Software IBM SPSS version 28.0 (IBM, Armonk, New York, USA). The analysis involved univariate, bivariate, and multivariate logistic regression. For the bivariate analysis, a chi-square test was used to examine the differences between caries scores and the hypothesized independent variables, which included sociodemographic factors, oral health practice, and general health behavior. Multivariate logistic regression was then performed to estimate the odds ratio (OR) and their 95% confidence intervals (95%CI) for caries. The chi-square an logistic regression analyses used a cut-off score based on the median DMFT score, categorizing them into 0-6 (low caries), and greater than 6 (high caries). In this analysis, the dependent variable for the multivariate regression was “caries”, as it was the outcome or response variable that the model was trying to predict or explain. The categorization of independent variables followed the categories established in the 2018 National Health Survey data, except for age.

## Results

Table 1 presents the mean and standard deviation of the DMFT index. The mean and standard deviation values of DT, MT, FT, and DMFT were 5.21 (± 4.48), 3.31 (± 6.22), 0.04 (± 0.28), and 8.87 (± 8.08), respectively. The Mann-Whitney test revealed significant differences across all variable categories, indicating that there is a marked variation of dental caries experience across sociodemographic backgrounds and oral health practice. The highest average DMFT scores were observed among respondents aged 45-64 years (10.97 ± 8.34 ) and in those aged over 65 years (17.24 ± 10.52). Other significant factors included living in rural areas (9.01 ± 8.51), lacking formal education 11.91(± 9.53SD), consuming sweets at least once a day (DMFT index > 6), not brush one’s teeth regularly (18.49 ±12.13), and smoking every day (10.03 ± 8.7).

**Table 1:** DMFT index of the respondents and analysis of the differences between each category

Variables	DT		MT		FT		DMFT		p-value
	Mean	SD	Mean	SD	Mean	SD	Mean	SD	
Age (years)									
15-24	2.96	3.18	0.15	0.58	0.02	0.19	3.14	3.33	< 0.001*
25-44	4.86	4.39	1.35	2.60	0.04	0.31	6.25	5.45	
45-64	6.21	5.26	4.73	6.66	0.03	0.26	10.97	8.34	
>64	6.4	5.66	10.81	10.5	0.04	0.32	17.24	10.52	
Sex									
Male	5.31	5.22	3.42	6.49	0.03	0.24	8.77	8.55	< 0.001*
Female	5.14	4.57	3.25	6.03	0.04	0.30	8.43	7.73	
Area of residence									
Urban	4.95	4.60	3.12	5.82	0.06	0.35	8.14	7.61	< 0.001*
Rural	5.48	5.07	3.52	6.62	0.01	0.17	9.01	8.51	
Educational level									
No school	6.29	5.44	5.61	8.22	0.01	0.15	11.91	9.53	< 0.001*
Elementary	5.70	5.07	3.60	6.43	0.01	0.14	9.32	8.31	
High school	4.49	4.31	2.03	4.45	0.05	0.32	6.57	6.49	
College	3.93	3.80	2.59	4.75	0.16	0.59	6.69	6.33	
Visit to a dental professional in the past year									
Yes	5.10	4.00	3.52	5.38	0.12	0.00	8.75	7.00	< 0.001*
No	5.81	4.98	3.24	5.86	0.03	0.00	9.07	7.00	
Consumption of sweets									
More than once a day	5.27	4.82	3.21	6.07	0.04	0.30	8.52	7.90	< 0.001*
Once a day	5.28	4.76	3.28	6.09	0.04	0.29	8.60	8.00	
3-6 times a week	5.12	4.82	3.27	6.24	0.04	0.26	8.43	8.11	
1-2 times a week	5.19	4.86	3.27	6.20	0.03	0.26	8.50	8.03	
< 3 times a month	5.15	4.84	3.50	6.44	0.04	0.32	8.68	8.27	
Never	5.32	5.20	3.89	6.74	0.02	0.18	9.24	8.56	
Consumption of sugary beverages									
More than once a day	5.57	5.11	3.65	6.57	0.03	0.27	9.25	8.46	< 0.001*
Once a day	5.28	4.81	3.41	6.36	0.03	0.25	8.72	8.13	
3-6 times a week	4.91	4.69	2.83	5.72	0.03	0.27	7.77	7.70	
1-2 times a week	4.96	4.65	2.96	5.73	0.04	0.31	7.95	7.59	
< 3 times a month	4.83	4.61	3.15	5.99	0.05	0.36	8.03	7.69	
Never	4.79	4.59	3.49	6.31	0.05	0.29	8.32	7.95	
Daily toothbrushing									
Yes	5.17	4.76	2.88	5.42	0.04	0.28	8.09	7.52	< 0.001*
No	6.03	6.32	12.45	12.19	0.00	0.08	18.49	12.13	
Smoking									
Everyday	6.32	5.55	3.68	6.29	0.02	0.22	10.03	8.47	< 0.001*
Not everyday	5.49	4.98	3.35	6.08	0.04	0.31	8.89	8.11	
No	5.49	4.59	3.10	5.54	0.05	0.33	8.64	7.39	
Mean	5.21	4.84	3.31	6.22	0.04	0.28	8.87	8.08	

*Significant level p < 0.05, Mann-Whitney test.

Table 2 describes the distribution of DMFT respondents based on the cut-off median value to differentiate between individuals with low and high caries experience. The table shows that there is a higher percentage of DMFT > 6 in respondents aged 45 years and over (> 60%), in those with low formal education (>50%), among denture wearers (76.9%), in those who drank sugary beverages more than once a day (51.2%), in those who do not brush their teeth every day (73.8%), and in those who smoke every day (52.1%). The chi-square test showed significant differences in all these variable categories.

**Table 2:** Distribution of the DMFT index according to the median cut-off (0-6 and > 6) and analysis of the differences of each category.

Variable	n	%	DMFT				p-value
			0–6		> 6		
			n	%	n	%	
Age							
15–24	5,589	15.1	4,817	86.2	772	13.8	< 0.0001*
25–44	1,4373	38.7	8,752	60.9	5,621	39.1	
45–64	1,3476	36.4	4,951	36.7	8,525	63.3	
> 64	3,619	9.7	756	20.9	2,863	79.1	
Sex							
Male	1,5183	40.9	7,972	52.5	7,211	47.5	0.060
Female	2,1874	59.1	11,304	51.7	10,57	48.3	
Area of residence							
Urban	1,8772	50.7	9,905	52.8	8,867	47.2	0.004*
Rural	1,8285	49.3	9,371	51.2	8,914	48.8	
Educational level							
No school	8,16	22.0	3,034	37.2	5,126	62.8	< 0.0001*
Elementary	10,958	29.6	5,225	47.7	5,733	52.3	
High school	15,673	42.3	9,671	61.7	6,002	38.3	
College	2,266	6.1	1,346	59.4	920	40.6	
Visit to a dental professional in the past year (times)							
Never	3,1292	84.4	16,492	52.7	14,8	47.3	< 0.0001*
1–3	4,297	11.6	2,03	47.2	2,267	52.8	
4–6	1,1	3.0	564	51.3	536	48.7	
> 7	368	1.0	190	51.6	178	48.4	
Consumption of sweets							
More than once a day	6,168	16.6	3,143	51.0	3,025	49.0	0.002*
Once a day	7,261	19.6	3,752	51.7	3,509	48.3	
3–6 times a week	7,912	21.4	4,228	53.4	3,684	46.6	
1–2 times a week	9,794	26.4	5,126	52.3	4,668	47.7	
< 3 times a month	3,929	10.6	2,056	52.3	1,873	47.7	
Never	1,993	5.4	971	48.7	1,022	51.3	
Consumption of sugary beverages							
More than once a day	11262	30.4	5,492	48.8	5,77	51.2	< 0.0001*
Once a day	10560	28.5	5,395	51.1	5,165	48.9	
3–6 times a week	5363	14.5	3,036	56.6	2,327	43.4	
1–2 times a week	5677	15.3	3,079	54.2	2,598	45.8	
< 3 times a month	2193	5.9	1,207	55.0	986	45.0	
Never	2002	5.4	1,067	53.3	935	46.7	
Daily toothbrushing							
Yes	35,378	95.5	18,836	53.2	16,542	46.8	<0.0001*
No	1679	4.5	440	26.2	1,239	73.8	
Smoking							
Everyday	9,356	25.3	4,483	47.9	4,873	52.1	< 0.0001*
Not everyday	2,972	8.0	1,582	53.2	1,39	46.8	
No	24,729	66.7	13,211	53.4	11,518	46.6	
TOTAL	37,057	100.0	19,276	52	17,781	48	

*Significant level p < 0.05, Chi-square test.

Table 3 presents the logistic regression analysis results for the caries score, with scores categorized into two groups: 0-6 (low caries) and greater than 6 (high caries), based on the median cut-off value. Respondents aged 65 years and over were 19.51 times more likely to experience higher caries {OR = 19.51, (95%CI (17.33–21.96), p < 0.05}. Male respondents had a 74% higher risk of caries than females {OR = 0.74, (95%CI (0.69–0.79)}, p < 0.05). Participants who live in rural areas had a 94% risk of caries {OR = 0.94, (96%CI (0.89–0.98) p < 0.05}. Those without formal education had a 1.62 times higher risk of experiencing caries {OR = 1.62 (95%CI (1.45–1.79) p < 0.05}. Those who had not visited a dentist in the past year were 78% more likely to experience caries {OR = 0.78 (95%CI (0.62–0.98) p < 0.05}. Respondents who ate sweets and drank sugary beverages once or more a day had 1.18 and 1.37 times higher chance to experience caries {OR = 1.18 (95%CI (1.05–1.33) p < 0.05} and {OR = 1.37 (95%CI (1.23–1.54) p < 0.05}. Participants who did not brush their teeth daily had a 1.48 times higher risk of experiencing caries {OR = 1.48 (95%CI (1.31–1.68) p < 0.05}. Respondents who smoked daily had a 1.3 times risk of experiencing caries {OR = 1.30 {95%CI (1.21–1.44) p < 0.05}. The logistic regression results indicated a good fit for the model, as evidenced by a Hosmer and Lemeshow test value of 0.268. Furthermore, the Nagelkerke R Square value was 0.218, suggesting that the model accounted for approximately 21.8% of the variability in the caries score.

**Table 3:** Multivariate logistic regression analysis for variables that influence caries in adults based on risk factors

Variable	OR	95% CI		p-value
		Lower	Upper	
Age				
15–24 (ref)				
25–44	3.79	3.48	4.13	< 0.001
45–64	9.64	8.83	10.63	< 0.001
> 64	19.51	17.33	21.96	< 0.001
Sex				
Male	0.74	0.69	0.79	< 0.001
Female (ref)				
Area of residence				
Urban (ref)				
Rural	0.94	0.89	0.98	0.012
Educational level				
No school	1.62	1.45	1.79	< 0.001
Elementary	1.51	1.36	1.67	< 0.001
High school	1.27	1.15	1.40	< 0.001
College (ref)				
Visit to a dental professional in the past year				
Never	0.78	0.62	0.98	0.03
1–3 times	1.07	0.84	1.35	0.56
4–6 times	0.87	0.67	1.13	0.37
> 7 times (ref)				
Consumption of sweets				
> 1 time a day	1.18	1.05	1.33	0.01
One time a day	1.11	0.99	1.25	0.06
3–6 times a week	1.074	0.95	1.20	0.22
1–2 times a week	1.011	0.90	1.12	0.84
< 3 times a month	0.93	0.82	1.05	0.28
Never (ref)				
Consumption of sugary beverages				
More than once a day	1.37	1.23	1.54	< 0.001
Once a day	1.28	1.15	1.43	< 0.001
3–6 times a week	1.17	1.04	1.32	1.01
1–2 times a week	1.23	1.09	1.38	< 0.001
< 3 times a month	1.05	0.92	1.21	1.44
Never (ref)				
Daily toothbrushing				
Yes (ref)				
No	1.48	1.31	1.68	<0.001
Smoking				
Everyday	1.30	1.21	1.44	<0.001
Not everyday	1.14	1.04	1.25	0.01
No (ref)				

## Discussion

This study reveals that the national average DMFT index in the adult population was 8.87, a figure that is considered moderate by the World Health Organization.^19^ The average DT (decayed teeth) score surpasses both the MT (missing teeth) and FT (filled teeth) scores, evincing that many respondents have untreated dental caries.

The highest DMFT index was observed among older adults. A systematic review of caries status in this population revealed that untreated caries remains a significant issue, with half or more of the older population affected.^20^ This prevalence rate varies across continents, with Asia and Africa recording the highest rates and Australia the lowest one. Globally, the median number of teeth with untreated caries is 1.55 per older adult.^20^ The incidence of caries is relatively higher in older adults for several reasons.^21^ One key factor is the change in dietary patterns due to decreased masticatory function. As the sense of taste and muscle strength decrease with age, older adults often struggle to chew hard-textured food. Consequently, they opt for softer, easier-to-chew, albeit often stickier foods that taste better but contain higher glucose levels.^21^ Another contributing factor is the decrease in salivary flow often observed in older adults.^22^ Reduced salivary flow can lead to food retention in the mouth, creating a conducive environment for bacterial growth, thus contributing to caries development.^22^ These shifts in dietary habits and physiological changes significantly contribute to the occurrence of caries in older adults.

Our analysis of the data also found that the respondents living in rural areas experienced a higher incidence of caries and even higher rates of untreated caries. This disparity could be attributed to the uneven distribution of dentists in Indonesia, with many rural areas still lacking adequate health services. Poor access to dental health services remains a significant concern for the Indonesian government, which is implementing various programs to ensure equitable distribution of dentists in all regions.^23^ Additionally, access to healthcare facilities is also influenced by Indonesia’s diverse geographical areas and uneven population distribution, with health service facilities predominantly established in densely populated areas.

The study also revealed that respondents with low formal education were at a higher risk of caries, with 62.8% of such respondents having a DMFT index greater than 6. The association between educational level and caries incidence could be due to inadequate knowledge regarding oral health, which eventually influences health behavior.^24^ Similar findings have been reported by studies conducted in Ethiopia, Paraguay, and by a previous study in Indonesia, in which the level of individual education affected the severity of caries in adults. ^3,15,25^

Excessive sugar intake, a known etiologic factor for caries, was also observed in our study. Sugar is metabolized by bacteria and produces acid, which lowers the pH of the oral cavity and triggers demineralization.^26^ Our study found that respondents who consumed sugary drinks and sweets once a day or more had a higher incidence of caries. This finding is in line with two previous studies in two cities in Indonesia, namely Semarang and Jakarta.^14,15^

Regular toothbrushing was identified as a protective factor for caries in our study. Most toothpastes contain fluoride, which protects the enamel against demineralization.^26^ Trials involving young permanent teeth have demonstrated this benefit of fluoride, showing a 20–30% decrease in caries prevalence in the population that uses fluoridated toothpaste.^27^

Smoking was also found to influence caries occurrence. Data show that among those who smoke daily, 52.1% had a higher caries experience. Studies show that cigarette smoke condensate promotes the adhesion of S mutans and Candida albicans to orthodontic materials.^28^ Another study found significant growth of S sanguis and S mutans strains after their incubation in atmospheric air, carbon dioxide, and cigarette smoke.^29^ S. mutans has been identified as the primary pathogen causing caries because of its acid-resistant, acidogenic, and strong biofilm-forming properties.^29^ These observations may explain our study findings that respondents with daily smoking habits have higher caries scores. A study of 33–34-year-olds in Indonesia previously found similar results. ^15^

This study also indicated that regular visits to the dentist are a protective factor against the risk of caries. However, 84.4% of respondents stated that they had not visited a dentist in the past year. This finding is similar to reports from Saudi Arabia, where only 6.4% of participants regularly visited a dentist.^30^ Adult dental service users are classified into routine attenders and problem-oriented attenders. The study results show routine attenders have better oral health and lower caries rates due to their frequent exposure to oral health care and guidance from dentists, which contributes to good self-care and plaque control.^31^

The logistic regression analysis in this study revealed that the examined variables accounted for 21.8% of the variation in the incidence of caries in the adult population of Indonesia. This suggests that while these variables play a role, a significant proportion of the variation (78.2%) is still unexplained, indicating the influence of other factors not included in the model. Therefore, further investigation into other theoretically relevant variables that could influence the development of caries is needed. Such a comprehensive approach could potentially provide a more holistic understanding of the factors contributing to caries occurrence.

Based on the findings of this study, a coordinated effort is required across various sectors - individuals, private entities, and government - to alleviate the impact of caries across all age groups.^32^ The significance of enhancing knowledge and self-efficacy through health education in schools cannot be overemphasized. Such an initiative necessitates a partnership between the Ministry of Education and the Ministry of Health to develop a synergistic school curriculum that integrates dental care into life skills education to enhance oral health literacy. As for dental practitioners, they should be more aware of early detection, prevention, and treatment of caries so that the morbidity rate associated with caries can be reduced.^33^ Furthermore, the government has a crucial role to play in ensuring equitable distribution of dental professionals, particularly in rural areas. This will enable residents in these areas to have better access to dental services.

A limitation of the present study lies in its cross-sectional design, which inherently prevents the determination of caries incidence, progression, and causality. Furthermore, not all potential contributing variables to caries, such as economic status, intraoral conditions such as the oral hygiene index, underlying or systemic diseases, and levels of S. mutants and lactobacilli in saliva, were measured. Despite these limitations, the strength of this study lies in its use of data from a national health survey, providing a representative snapshot of the oral health status of the Indonesian population.

For a more thorough understanding of the factors causing caries, an in-depth, longitudinal study is recommended. This approach could offer precise insights into the dynamics of caries development, aiding in the formulation of effective population-based oral health strategies in Indonesia. However, such studies come with their own set of challenges, such as managing resource allocation, maintaining participant engagement, and overcoming logistic difficulties. Overcoming these obstacles would require a meticulous planning approach, synergistic partnerships, and innovative methodologies.

## Conclusion

This study sought to shed some light on the multifaceted nature of caries occurrence in the Indonesian adult population, highlighting the influence of various factors, ranging from lifestyle habits and oral hygiene practices to access to dental services, age, and educational level. Despite these limitations, the study could provide a snapshot of oral health in Indonesia, focusing on untreated caries prevalence. The findings call for a multisectoral approach to mitigate caries impact, including enhancing oral health literacy, promoting early detection, and ensuring equitable distribution of dental services.
